# Effects of a Structured Physical Activity Program on Quality of Life in Older Adults: A Quasi-Experimental Study

**DOI:** 10.3390/healthcare14050685

**Published:** 2026-03-09

**Authors:** Evgenia Kouli, Evangelos Bebetsos, Maria Michalopoulou, Filippos Filippou

**Affiliations:** Department of Physical Education and Sport Science, School of Physical Education, Sport Science and Occupational Therapy, Democritus University of Thrace, 69100 Komotini, Greece

**Keywords:** quality of life, elderly, physical activity, structured program

## Abstract

Background/Objectives: Quality of life is conceptualized as a multidimensional construct encompassing subjective well-being, health, and social functioning. Evidence suggests that engagement in physical activity contributes to higher quality of life scores among older adults, indicating that structured exercise programs can positively influence both physical and psychological domains in this population. The present study examined the impact of an 18-week structured physical exercise program on the quality of life of older adults, assessed through the World Health Organization Quality of Life-BREF (WHOQOL-BREF) instrument, which consists of four domains: physical health, psychological, social relationships and environment. A total of 86 participants were allocated to three groups: individual exercise (*n* = 31), collaborative exercise (*n* = 32), and a control group (*n* = 23). Quality of life was evaluated before and after the intervention using the WHOQOL-BREF. Results: Correlation analysis indicated strong relationships among the WHOQOL-BREF domains, both before and after the program. Repeated-measures analysis revealed no significant Group × Time interaction effects for any WHOQOL-BREF domain. A significant main effect of Time was observed for the Environment domain, indicating a small overall decrease across all groups during the study period. Conclusions: The structured exercise protocol did not lead to greater changes in quality of life compared to the control condition. Perceived environmental quality of life showed a small overall decrease over time across participants.

## 1. Introduction

Population aging is rapidly increasing worldwide, resulting in a larger proportion of older adults experiencing significant physical and psychological challenges that can negatively affect their well-being and quality of life [1,2]. Psychological conditions such as loneliness, depression, anxiety, and cognitive decline frequently remain undiagnosed until they substantially hinder daily functioning, threatening independence and social participation [3,4]. Quality of life (QoL) in later life is recognized as a multidimensional construct that includes perceptions of physical functioning, psychological well-being, social relationships, and environmental support [5,6,7]. Consequently, the promotion of strategies that protect physical and mental health, support autonomy, and foster social inclusion is essential to improve healthy aging outcomes.

Regular participation in physical activity is widely supported as an effective, low-cost, and socially accessible means to mitigate age-related physiological decline and enhance QoL [2,8]. Exercise strengthens the musculoskeletal and cardiovascular systems, improves balance and mobility, supports cognitive functioning, and reduces symptoms of depression and anxiety [9,10,11]. Importantly, group-based exercise programs also offer opportunities for social interaction, emotional expression, and reduced feelings of isolation, which further contribute to improved psychological and social well-being [12]. Evidence indicates that older adults who engage in regular physical activity report higher QoL scores than their sedentary peers across all primary domains, even when controlling for age-related health changes [2,13,14].

Intervention studies reinforce these observations. Strength training and multi-component exercise programs have demonstrated improvements in physical functioning, autonomy, vitality, and social participation, thereby enhancing overall QoL in community-dwelling older adults [15,16]. Exercise modes may also produce domain-specific QoL benefits. For instance, strength training is strongly associated with improved physical functioning, whereas swimming and other aerobic activities appear to enhance psychological and social dimensions of QoL to a greater extent [17,18]. Collectively, such findings highlight the importance of tailoring exercise programs to the functional needs and preferences of older individuals to maximize health outcomes.

Additional research suggests that physical activity supports health behaviors that help limit disability and reduce premature mortality and healthcare use [19,20,21]. Even low-intensity daily activities have been associated with increased satisfaction with health, reduced medication use, and improved QoL in physical, psychological, and environmental areas [22]. Furthermore, older adults who participate in structured programs—particularly those with cognitive engagement or social components—show enhanced physical function and better subjective well-being than those exercising independently or less frequently [23,24,25]. Reducing sedentary time and replacing it with moderate activity has also been shown to benefit all QoL domains [26,27,28], reinforcing activity promotion as a critical public health objective for aging populations.

Nordic walking-based interventions further exemplify how structured physical activity can restore mobility, reduce pain, improve mood, and enhance health perceptions among older adults [29]. A year-long Nordic walking program resulted in significant QoL improvements in physical functioning, vitality, mental health, and social functioning compared with inactive controls, demonstrating that participation in supervised exercise may help counteract the main barriers to physical activity such as fear of falling, lack of motivation, and low social support [30,31]. These findings emphasize the added value of guidance, structured exercise, and group dynamics in facilitating adherence and optimizing QoL outcomes.

Functional fitness also plays an integral role in quality of life. Declines in standing balance, gait speed, and muscle strength predict disability, frailty, and increased healthcare needs [32,33]. Individuals with limited physical performance demonstrate reduced independence, poorer QoL, and higher risk of adverse events, making early screening and intervention essential in geriatric health care [3,4,34]. By contrast, older adults with higher levels of physical activity maintain autonomy longer and are more likely to experience successful aging outcomes [35,36].

Taken together, previous evidence consistently demonstrates that physical activity—particularly when structured and socially supported—can improve health-related quality of life in older adults across physical, psychological, and environmental dimensions. However, the degree to which different exercise contexts (e.g., individual vs. group-based participation) influence changes in QoL, remains insufficiently explored. Given that environmental perceptions and opportunities for social engagement contribute substantially to subjective well-being in late life, further research is needed to clarify how exercise settings shape QoL responses.

Although the positive association between physical activity and quality of life in older adults is well documented, less attention has been given to how quality-of-life domains respond over time within real-world community settings and across different participation formats. In particular, the environment domain—which reflects perceptions of safety, accessibility, and opportunities for engagement—has been relatively underexplored in exercise-related research. The present study addresses this gap by examining changes over time in quality of life among Greek community-dwelling older adults participating in an 18-week structured exercise program delivered in individual and collaborative formats.

Therefore, the present study examined the effects of an 18-week structured physical exercise program—delivered either individually or collaboratively—on the quality of life of older adults, using the WHOQOL-BREF’s four domains (physical health, psychological health, social relationships, and environment). This approach may offer new insights into how exercise format influences perceived quality of life, particularly regarding environmental support and social connectedness, which are increasingly recognized as key components of aging well.

## 2. Materials and Methods

### 2.1. Research Design

The study employed a quasi-experimental design comprising two exercise conditions (individual and collaborative) and one control condition without intervention. Participation in the intervention was non-randomised and based on enrolment in the community programs (self-selected). Allocation to exercise format within the intervention group was determined by the researchers, and the control group comprised community-dwelling older adults who did not enroll in the exercise programs. No a priori sample size calculation was performed; sample size was determined by participant availability within the community program. The dependent variables consisted of the four domains assessed by the questionnaire, measured both before and after the intervention. Ethical approval for the study was obtained from the University’s Ethics and Deontology Committee prior to its commencement (approval number 6118/29 (28 September 2022)).

### 2.2. Participants and Procedure

The study sample comprised 86 participants aged between 60 and 89 years (M = 70.02, SD = 6.54). The majority of participants were women (74.4%), while men constituted 25.6% of the total sample. Regarding program type, 36.0% of participants engaged in an individual exercise program, 37.2% participated in a collaborative program, and 26.7% did not take part in any intervention. In terms of educational background, the largest proportion were primary school graduates (37.2%), followed by high school graduates (20.9%) and university degree holders (19.8%). With respect to marital status, most participants were married (65.1%) or widowed (26.7%), whereas smaller percentages were single (4.7%) or divorced (3.5%). Additionally, 38.1% of participants reported the presence of a health condition, while 61.9% indicated they did not.

The intervention program spanned 18 weeks and consisted of two one-hour sessions per week at a moderate intensity level. Its design was based on functional training principles for older adults, emphasizing the preservation of musculoskeletal health and the prevention of falls—both crucial components for sustaining quality of life in later adulthood.

Participants allocated to the exercise conditions comprised all older adults who enrolled in the physical activity programs offered by the two Open Care Centers during the study period (individual exercise: *n* = 31; collaborative exercise: *n* = 32). As no additional participants were available for enrollment in the exercise programs during this period, the control group was formed by recruiting older adults from the same community who did not participate in organized physical activity. No a priori sample size calculation was performed, as the study included the full population of available program participants. In terms of group allocation, 31 participants were assigned to the individual exercise program, 32 to the collaborative exercise program, and 23 to the control group, which did not participate in any exercise activity. Participants enrolled in the study based on availability for specific program time slots. Assignment to the individual or collaborative exercise format was determined by the researchers and was not chosen by participants. The control group consisted of older adults who did not attend the Open Care Centers and therefore did not participate in any structured exercise program. Although participation in the intervention was non-randomized, this approach reflects real-world conditions in community-based settings.

Participants were assigned identification codes upon enrollment. All questionnaires were completed anonymously, and data were entered and analyzed using coded datasets without access to identifiable participant information. Due to the nature of the intervention, participant blinding was not feasible. Outcome assessment relied on standardized self-report questionnaires administered under identical conditions for all participants.

### 2.3. Instruments

The questionnaire used to measure Quality of Life was the World Health Organization Quality of Life–BREF (WHOQOL-BREF). It consists of 26 self-report items rated on a five-point Likert scale. Domain scores were computed in accordance with the WHOQOL-BREF scoring manual. Negatively phrased items were reverse-coded prior to calculation. For each domain, item responses were averaged to obtain mean-item scores ranging from 1 (not at all) to 5 (completely), with higher scores indicating better perceived quality of life. In line with WHO guidance, domain scores were not transformed to the 0–100 scale; raw mean-item scores were retained for analysis to facilitate interpretability and consistency across measurement points. The items are organized into four domains: physical health, psychological health, social relationships, and environment, along with two general items assessing overall quality of life and general health. For example, the WHOQOL-BREF includes items such as: “To what extent do you feel that physical pain prevents you from doing what you need to do?” (Physical Health), “How much do you enjoy life?” (Psychological), “How satisfied are you with your personal relationships?” (Social Relationships), and “How satisfied are you with the conditions of your living place?” (Environment). The instrument was developed by the WHOQOL Group (1998) and has been extensively validated across different populations and cultural contexts. The Greek version of the WHOQOL-BREF (26 items) has demonstrated good reliability and validity in Greek populations. Previous studies have reported acceptable to good internal consistency, with Cronbach’s alpha coefficients ranging from 0.67 to 0.81 across the four domains, supporting the reliability of the instrument. In addition, evidence of construct and convergent validity has been documented through significant correlations with established measures of health status and psychological well-being. The Greek WHOQOL-BREF has also been applied in studies involving older adults and physical activity interventions, confirming its suitability for assessing quality of life outcomes in aging and exercise-related research [37,38].

### 2.4. Intervention

The intervention spanned 18 weeks and consisted of two 60-min sessions per week, carried out at a moderate intensity. Each session followed a standardized structure, beginning with a 5–10-min warm-up that included light walking, mobility drills, and stretching exercises. The central component of the session (approximately 40 min) emphasized strength training for the major muscle groups, complemented by static and dynamic balance activities. Sessions concluded with a 10–15-min cool-down phase involving stretches for the engaged muscles and breathing exercises designed to promote relaxation. The program was developed and implemented in accordance with international recommendations for physical activity in older adults, with the primary aim of improving both functional fitness and psychological well-being.

At the initial meeting, participants were informed that their participation was voluntary. Before beginning the program, they were required to provide written informed consent after receiving detailed information regarding the procedures, potential benefits, and possible risks of involvement. In addition, medical clearance from a cardiologist confirming their ability to safely participate in the intervention was obtained. Outcome measures were collected at baseline and at the completion of the 18-week intervention for all study groups.

The exercise protocol incorporated moderate-intensity aerobic activity as well as resistance training using body weight, dumbbells, and everyday household objects, thereby equipping participants with exercises they could continue independently at home after the program. In addition, balance, flexibility, and coordination activities were included to support fall prevention.

In the collaborative format of the program, several exercises were carried out in pairs, small groups, or circle formations to highlight cooperation in achieving shared goals. For balance tasks, participants supported one another rather than relying on chairs or fixed equipment. Strength exercises were also structured for partnership, such as performing assisted squats with a partner, in order to enhance safety and minimize the risk of falls. Attendance was monitored throughout the intervention period. All exercise sessions were supervised by trained instructors and followed a standardized protocol to ensure intervention fidelity.

### 2.5. Data Analysis

Baseline differences were assessed descriptively using group means, percentages, and standardized mean differences (SMD). To investigate the relationship between the domains of Quality of Life questionnaire, Pearson correlation analysis was used, with the level of statistical significance set at *p* < 0.05.

Repeated-measures ANOVA (Time × Group) was conducted for each WHOQOL-BREF domain and the two general items (overall quality of life and overall health) to evaluate the main effects of time and Group × Time interaction effects. There was one independent factor: group (individual, collaborative and control group) and one dependent factor: measurement (before and after intervention). Accordingly, all statistical analyses focused on baseline and post-intervention measurements.

Repeated-measures ANOVA was selected as the primary analytic approach because the study involved two measurement occasions, complete data across time points, and acceptable baseline comparability across groups. Under these conditions, repeated-measures ANOVA provides a suitable method for examining main effects of time and group-by-time interactions. Given the non-randomized design, covariate-adjusted ANCOVA models were additionally conducted as sensitivity analyses.

Analyses were conducted using data from participants who completed both pre- and post-intervention assessments; no imputation of missing data was performed, reflecting a per-protocol analytical approach.

## 3. Results

Primary analyses used repeated-measures ANOVA; covariate-adjusted ANCOVA models were conducted as sensitivity analyses.

Attendance was monitored throughout the 18-week intervention. Of the 63 participants allocated to the exercise conditions, 46 participants (73.0%) attended more than 70% of the 36 planned sessions. Specifically, 21 participants from the individual exercise group and 25 participants from the collaborative exercise group met this adherence threshold. Adherence levels were comparable between the two exercise formats. Of the 86 enrolled participants, repeated-measures analyses were conducted using complete-case data for each domain (participants with both baseline and post-intervention responses). As a result, the analytic sample size varied across domains.

### 3.1. Differences on Initial Measurements

Baseline analyses indicated no statistically significant differences among the three groups on demographic characteristics or WHOQOL-BREF domain scores. Standardized effect sizes were not statistically significant, suggesting acceptable baseline comparability.

*Baseline comparability*

Baseline demographic characteristics and WHOQOL-BREF domain scores for all groups are presented in Table 1. Given the quasi-experimental design, baseline balance was assessed descriptively using group means, percentages, and standardized mean differences (SMD). The observed SMD values were small, indicating negligible group differences and supporting baseline comparability across study groups.

### 3.2. Correlations Among WHOQOL-BREF Domains

Pearson correlation analyses were conducted to explore the interrelations among the four WHOQOL-BREF domains and the two general items (overall quality of life and overall health perception), before and after the intervention. The pre-intervention results (Table 2) revealed a pattern of significant positive associations across all domains. Specifically, overall perception of quality of life was moderately and significantly related to overall perception of health (r = 0.286, *p* < 0.01), as well as to the physical (r = 0.395, *p* < 0.01), psychological (r = 0.426, *p* < 0.01), social relationships (r = 0.474, *p* < 0.01), and environment domains (r = 0.456, *p* < 0.01). Notably, strong interconnections were observed among the physical, psychological, and environmental domains, indicating that individuals reporting higher physical well-being also tended to experience greater psychological and environmental satisfaction.

Following the intervention (Table 3), this positive interdependence among domains was largely maintained, with several correlations becoming stronger. Overall perception was significantly associated with overall health perception (r = 0.379, *p* < 0.01), as well as with physical (r = 0.446, *p* < 0.01), psychological (r = 0.549, *p* < 0.01), social relationships (r = 0.490, *p* < 0.01), and environment (r = 0.629, *p* < 0.01). The particularly strong link between overall perception and the environment domain suggests that post-intervention, participants’ general quality of life was more closely aligned with their sense of environmental support and comfort. These consistent and positive associations across measurement points highlight a stable and interconnected structure of quality of life dimensions.

### 3.3. Differences Between Measurements and Groups

A repeated-measures analysis was conducted to examine potential differences across the two measurement points for each group and to test for group × time interactions across the WHOQOL-BREF domains. As shown in Table 4, no significant interaction effects were observed for any of the domains, indicating that changes over time were comparable across groups. Mean scores for overall quality of life and overall health perception remained stable between the first and second measurements, with minimal variation across groups. Physical and psychological domains showed small, non-significant decreases over time, while social relationships remained stable. In contrast, a significant main effect of time was found for the environment domain (F(1,58) = 12.31, *p* < 0.001, *ηp*^2^ = 0.18), reflecting an overall decrease from baseline (M = 3.89, SD = 0.49) to post-intervention (M = 3.73, SD = 0.49). This finding indicates that, irrespective of group allocation, participants reported slightly lower environmental quality of life at post-intervention compared with baseline. The above results are presented in detail in Table 4.

### 3.4. Sensitivity Analysis: Covariate-Adjusted ANCOVA

To account for the non-randomized design, covariate-adjusted ANCOVA models were conducted for each domain. Post-intervention scores were entered as dependent variables, baseline scores as covariates, and program group as a fixed factor. Results are presented in Table 5.

The adjusted analyses were largely consistent with the repeated-measures ANOVA findings, showing no statistically significant group effects for Overall Quality of Life, Overall Health Perception, Psychological Health, Social Relationships, or Environment.

A modest adjusted group effect was observed for Physical Health (F(2,57) = 3.471, *p* = 0.038, *ηp*^2^ = 0.109); however, this was not reflected in the primary Group × Time interaction analysis and should therefore be interpreted cautiously.

## 4. Discussion

The present study examined changes in quality of life among older adults participating in an 18-week structured exercise program delivered in individual and collaborative formats within a community setting. Contrary to expectations derived from prior intervention research, no significant Group × Time interaction effects were observed across any WHOQOL-BREF domain. This indicates that the exercise intervention did not produce superior changes in quality of life compared to the control group. Participation in the intervention was based on enrollment in community programs rather than random assignment. Although allocation to exercise format was determined by the researchers, overall group allocation was non-randomized, reflecting real-world community conditions.

A significant main effect of Time was identified only for the Environmental domain, reflecting a small overall decrease from baseline to post-intervention across groups. Therefore, this change cannot be attributed specifically to the exercise protocol implemented in this study. The covariate-adjusted ANCOVA analyses were largely consistent with these findings and did not indicate significant adjusted group effects for the Environment domain.

Consistent with this, the Group × Time interaction for the Environment domain was not significant, indicating that the temporal decrease did not differ between the intervention and control groups.

A modest group effect emerged for the Physical Health domain after controlling for baseline scores. However, this effect was not observed in the primary interaction analysis and should therefore be interpreted cautiously. Given the quasi-experimental design and the absence of consistent effects across domains, this isolated adjusted finding does not alter the overall conclusion of the study.

This null finding warrants careful consideration. Several factors may help explain the lack of differential effects. First, the moderate intensity and twice-weekly frequency of the program may have been insufficient to elicit measurable physiological or psychological changes within 18 weeks. Second, participants reported moderate-to-high baseline quality-of-life scores (approximately 3.7–4.0 out of 5), suggesting a potential ceiling effect that limited observable change. Third, the control group consisted of community-dwelling older adults who may have remained socially and physically active during the study period, thereby attenuating between-group differences. The relatively stable scores across the physical health, psychological, and social domains may reflect the moderate baseline levels observed in this sample, leaving limited room for detectable short-term change and warranting domain-specific interpretation [14,16]. Previous studies have reported improvements in subjective well-being and quality-of-life domains following structured physical activity interventions in older adults [23,24]; however, such effects were not observed differentially in the present study.

The absence of significant change in the social relationships domain warrants careful interpretation. The WHOQOL-BREF social domain primarily assesses satisfaction with close personal and family relationships rather than broader social participation or informal social contact. Consequently, while group-based exercise may facilitate interaction, companionship, and a sense of belonging, these changes may not be adequately captured by this domain. Moreover, participants were community-dwelling older adults already engaged in social environments, which may have resulted in relatively high baseline social support and limited the potential for further measurable change. In addition, social interaction may not be uniformly perceived as beneficial by all older adults. Previous research suggests that individuals with depressive symptoms or psychological vulnerability may experience social engagement as demanding rather than supportive, which can influence perceptions of social well-being and quality of life [11].

Although changes in the environment domain cannot be attributed specifically to the exercise intervention, these shifts over time may reflect indirect influences associated with participation in a structured study context. Although this change cannot be attributed specifically to the exercise intervention, it may reflect indirect effects associated with participation in a structured study context. Improved physical function and mobility may enhance older adults’ confidence in navigating their surroundings, which may influence perceptions of access to leisure opportunities, services, and community resources [31,35]. In addition, repeated exposure to organized settings and routines may influence perceptions of safety, familiarity, and environmental support, as suggested in previous studies examining structured physical activity and quality-of-life perceptions in older adults [23,30]. Together, these factors may help explain observed changes in environmental quality of life over time among community-dwelling older adults.

The strong and consistent correlations observed among quality of life domains both before and after the intervention further underscore the multidimensional and interconnected nature of well-being in older age. In particular, the strengthened association between overall quality of life perception and the environment domain post-intervention suggests that environmental support may play an increasingly central role in how older adults evaluate their overall well-being. This finding is consistent with prior evidence emphasizing the importance of supportive environments and accessible opportunities for physical activity in promoting successful aging [35,36].

Contrary to expectations derived from earlier studies highlighting the psychosocial benefits of group-based exercise [12], collaborative participation did not produce superior outcomes compared to individual exercise. This finding may reflect comparable levels of engagement and perceived autonomy across participants in both exercise formats. Additionally, the relatively short duration and moderate intensity of the program may have limited domain-specific differentiation, particularly in psychological and social outcomes.

### 4.1. Limitations

The present study has several limitations that should be considered when interpreting the findings. First, allocation to intervention and control conditions was non-randomized, which may introduce selection bias and limit internal validity. Although baseline analyses indicated acceptable comparability across groups, unmeasured confounding cannot be excluded. Because participation in the intervention was self-selected through enrolment in community programs, differences in motivation, baseline health status, or lifestyle characteristics between groups may have influenced the observed outcomes. In addition, the absence of an a priori sample size calculation and the use of a per-protocol analytical approach should be considered when interpreting the findings. Also, the control group consisted of older adults who did not attend the Open Care Centers, and therefore differences between exercisers and non-exercisers may partly reflect contextual or lifestyle factors unrelated to the intervention itself. Finally, the relatively short intervention duration and reliance on self-reported quality-of-life measures may have limited the detection of domain-specific changes over time. Future studies employing randomized designs, longer follow-up periods, and objective outcome measures are warranted.

### 4.2. Practical Implications

From a practical perspective, the findings suggest that participation in structured community-based activities may be associated with changes in perceived environmental quality of life over time, although these changes were modest and not intervention-specific. This has important implications for community-based interventions, as it indicates flexibility in program design while supporting participation and accessibility. Given common barriers to participation—such as scheduling constraints, fear of falling, or preference for autonomy—offering multiple formats may help increase adherence and inclusivity [22,31].

Moreover, the observed interconnections among quality of life domains highlight the value of holistic program planning that considers not only physical fitness but also environmental accessibility, safety, and perceived support. Incorporating familiar spaces, clear routines, and adaptable equipment may further enhance environmental satisfaction and overall well-being. Health professionals and policymakers should therefore prioritize accessible, structured exercise opportunities as part of broader strategies aimed at promoting healthy and independent aging.

## 5. Conclusions

The present study contributes to the growing body of research examining quality of life in older adults participating in structured physical activity contexts. A small overall decrease in the environment dimension of quality of life was observed over time during the study period, while other domains remained stable. No differential effects were observed between individual, collaborative, and control groups, indicating that changes occurred irrespective of exercise format.

These findings reinforce the multidimensional nature of quality of life in later life and emphasize the importance of environmental perceptions as a key component of subjective well-being. Participation in organized activities within community settings may be associated with changes in older adults’ perceptions of environmental support, safety, and access to resources, thereby contributing to overall life satisfaction.

Future research should consider randomized designs, longer intervention periods, and additional follow-up assessments to clarify whether specific exercise contexts yield differential long-term benefits across quality of life domains. Nonetheless, participation in structured community-based activities may be associated with changes in perceived environmental quality of life over time, although such changes may be modest and not intervention-specific.

## Figures and Tables

**Table 1:** Baseline Characteristics of Participants by Group.

Variable	Individual (*n* = 31)	Collaborative (*n* = 32)	Control (*n* = 23)	SMD (Ind vs. Ctrl)	SMD (Collab vs. Ctrl)
Age (years)	70.74 ± 4.36	68.62 ± 6.27	71.00 ± 8.92	−0.04	−0.32
Sex, *n* (%)					
Men	5 (16.1%)	7 (21.9%)	10 (43.5%)	−0.59	−0.46
Women	26 (83.9%)	25 (78.1%)	13 (56.5%)	—	—
Education, *n* (%)				—	—
Primary	15 (48.4%)	10 (31.3%)	7 (30.4%)	—	—
Secondary	5 (16.1%)	12 (37.5%)	7 (30.4%)	—	—
Higher	6 (19.3%)	7 (21.9%)	9 (39.1%)	—	—
WHOQOL-BREF Domains (Baseline)					
Overall Quality of Life	3.97 ± 0.61	3.97 ± 0.54	3.78 ± 0.67	0.29	0.30
Overall Health Perception	3.77 ± 0.88	4.16 ± 0.81	3.70 ± 1.26	0.06	0.41
Physical Health	3.81 ± 0.64	3.92 ± 0.56	3.65 ± 0.91	0.20	0.30
Psychological	3.82 ± 0.46	3.86 ± 0.62	3.78 ± 0.63	0.07	0.13
Social Relationships	3.58 ± 0.68	3.73 ± 0.81	3.65 ± 0.73	−0.10	0.11
Environment	3.80 ± 0.54	3.83 ± 0.56	3.83 ± 0.53	−0.06	0.01

Note: Values are mean ± SD or *n* (%); SMD = standardized mean difference.

**Table 2:** Correlations, Mean and Standard Deviation of WHOQOL-BREF Domains at Baseline.

	1	2	3	4	5	6	M ± SD
Overall Quality of Life	1						3.92 ± 0.60
Overall Health Perception	0.29 **	1					3.90 ± 0.98
Physical Health	0.40 **	0.65 **	1				3.80 ± 0.70
Psychological	0.43 **	0.32 **	0.53 **	1			3.83 ± 0.56
Social Relationships	0.47 **	0.24 **	0.49 **	0.62 **	1		3.64 ± 0.74
Environment	0.46 **	0.32 **	0.61 **	0.57 **	0.58 *	1	3.82 ± 0.54

Note: * *p* < 0.05; ** *p* < 0.01.

**Table 3:** Correlations, Mean and Standard Deviation of WHOQOL-BREF Domains at Post-Intervention.

	1	2	3	4	5	6	M ± SD
Overall Quality of Life	1						3.96 ± 0.63
Overall Health Perception	0.38 **	1					3.93 ± 0.92
Physical Health	0.45 **	0.41 **	1				3.76 ± 0.70
Psychological	0.55 **	0.46 **	0.54 **	1			3.66 ± 0.83
Social Relationships	0.49 **	0.23	0.33 *	0.40 **	1		3.80 ± 0.51
Environment	0.63 **	0.20	0.54 **	0.47 **	0.43 **	1	3.73 ± 0.48

Note: * *p* <0.05; ** *p* < 0.01.

**Table 4:** Repeated Measures ANOVA Results for Quality of Life Domains (Before vs. After by Program Group).

Domain	Time (Mean ± SD) Before	Time (Mean ± SD) After	Time Effect F (1, df)	*p*	*ηp* ^2^	Time × Group F (2, df)	*p*	*ηp* ^2^
Overall QoL	3.97 ± 0.60	3.96 ± 0.63	0.10 (1,65)	0.753	0.00	0.71 (2,65)	0.50	0.02
Overall Health	3.99 ± 0.97	3.93 ± 0.92	0.11 (1,65)	0.742	0.00	1.25 (2,65)	0.29	0.04
Physical Health	3.85 ± 0.69	3.76 ± 0.71	2.29 (1,58)	0.136	0.04	2.14 (2,58)	0.13	0.07
Psychological	3.90 ± 0.55	3.81 ± 0.52	3.64 (1,62)	0.061	0.06	0.46 (2,62)	0.64	0.02
Social Relationships	3.68 ± 0.74	3.68 ± 0.81	0.03 (1,52)	0.858	0.00	1.42 (2,52)	0.25	0.05
Environment	3.89 ± 0.49	3.73 ± 0.49	12.31 (1,58)	<0.001	0.18	1.31 (2,58)	0.28	0.04

Note: Values are mean ± SD. *ηp*^2^ = partial eta squared. Analytic sample size varied by domain due to complete-case analysis (participants with both baseline and post-intervention data for the respective domain).

**Table 5:** Covariate-Adjusted ANCOVA Results (Post Score Adjusted for Baseline).

Domain	F(2,df)	*p*	Partial *η*^2^
Overall Quality of Life	1.59 (2,64)	0.212	0.05
Overall Health Perception	0.16 (2,64)	0.849	0.01
Physical Health	3.47 (2,57)	0.038	0.11
Psychological	0.64 (2,61)	0.533	0.02
Social Relationships	1.23 (2,51)	0.302	0.05
Environment	2.06 (2,57)	0.136	0.07

Note: Post-intervention scores were entered as dependent variables, baseline scores as covariates, and program group as a fixed factor.

## Data Availability

The data that support the findings of this study are available from the corresponding author upon reasonable request. Due to privacy and confidentiality considerations related to the study participants, the datasets are not publicly available.
